# Supplementary material for: Characterization of Regulatory T Cells in Patients Infected by Leishmania Infantum
Source: Trop Med Infect Dis. 2022 Dec 27;8(1):18. doi: 10.3390/tropicalmed8010018 (PMC9864225; doi:10.3390/tropicalmed8010018)
Supplement: Supplementary file 1 [file tropicalmed-08-00018-s001.zip › tropicalmed-2096657-supplementary.pdf]

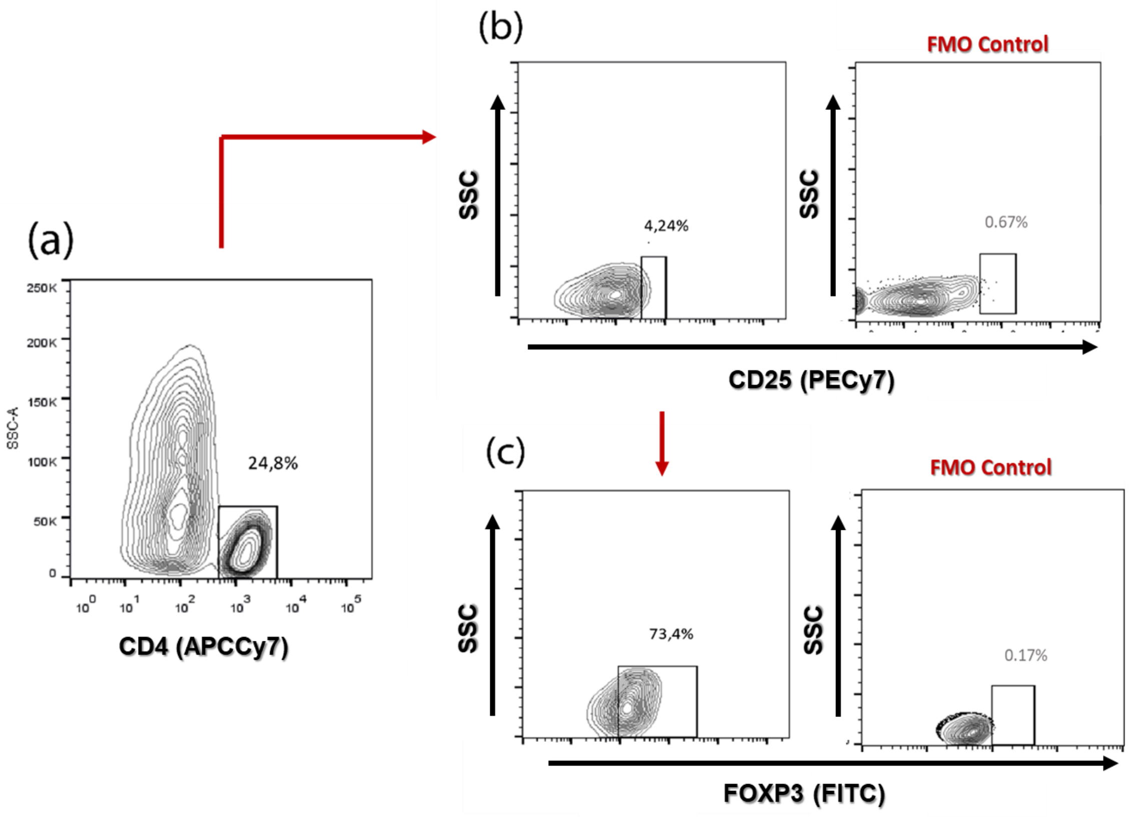

Figure S1. Representative analysis strategy for selecting Treg cells. (**a**) $CD4^+$ T lymphocyte gating based on the Side Scatter parameters–SSC and $CD4^+$. (**b**) $CD4^+CD25^{high}$ T cells using Side Scatter parameters–SSC × $CD25^+$ and CD25 FMO Control. (**c**) Selection of $CD4^+$ $CD25^{high}FOXP3^+$ T cells using the Side Scatter parameters–SSC × FOXP3 and FOXP3 FMO Control.
